# Trojan mosquitoes control dengue

**DOI:** 10.1038/s43856-021-00018-y

**Published:** 2021-07-20

**Authors:** Andreia Cunha

**Affiliations:** Communications Medicine, https://www.nature.com/commsmed

## Abstract

Dengue virus is transmitted by *Aedes aegypti* mosquitoes and causes the disease known as dengue. In a trial published in *The New England Journal of Medicine*, Utarini and colleagues report that release of wolbachia-infected *A. aegypti* populations in a dengue endemic area reduces the number of symptomatic cases and of hospitalisations.

**Figure:**
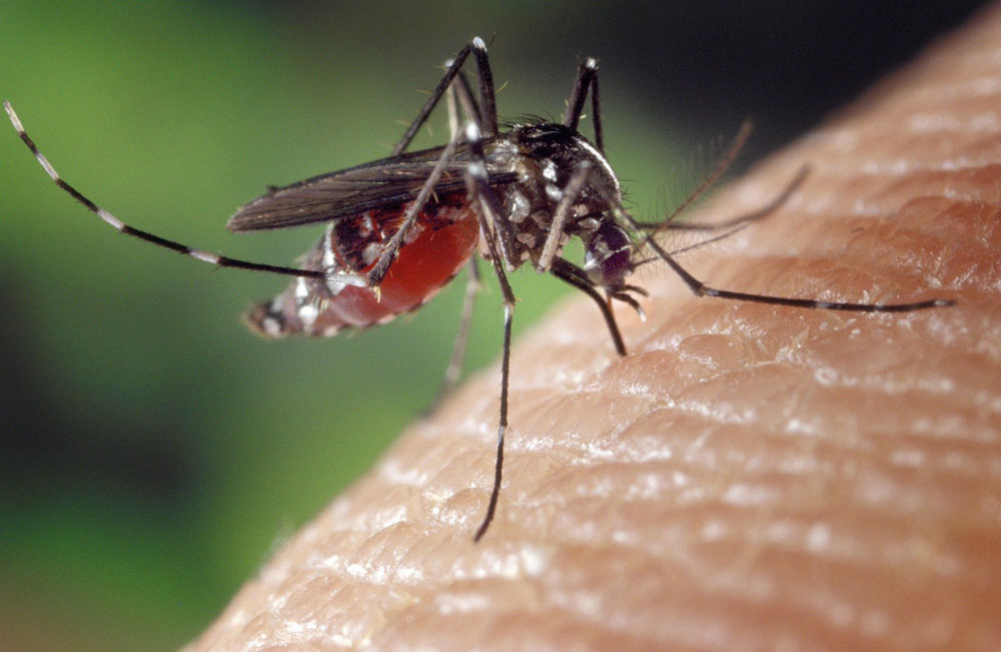
Pixabay

According to the World Health Organisation, dengue is estimated to affect 100–400 million people worldwide every year, putting considerable strain on health services in low- and middle-income countries. Global warming, among other factors, means that this *Aedes*-borne virus is emerging into new geographical areas and the incidence has increased significantly in the last decades globally, placing dengue as one of the top ten global health threats.

Since the four dengue virus serotypes are transmitted to humans mainly by *A. aegypti* mosquitoes, public health interventions focus on vector control measures. One possibility is to control infection of the vector, but this has so far not been trialled with enough success. It is known that introgression of *A. aegypti* with the bacteria *Wolbachia pipientis* confers resistance to infection of the mosquito with dengue virus. Utarini and colleagues have performed a cluster-randomised controlled trial to assess the efficacy of releasing *A. aegypti* mosquitoes, infected with the *w*Mel strain of *W. pipientis*, on reducing the incidence of dengue and hospitalisation from the disease, in the city of Yogyakarta in Indonesia^1^.

Between March and December 2017, 12 geographic clusters were randomly assigned to the intervention of releasing *w*Mel-infected mosquitoes, and twelve others to serve as controls in which no mosquitoes were released. Primary health care facilities in the trial area monitored patients for acute fever with no signs of other non-arboviral disease, and recruited individuals between 3 and 45 years old. The primary endpoint was virologically-confirmed dengue virus infection of any severity, and the safety end points included hospitalisation within 21 days of enrolment.

In total, 3721 participants were recruited in intervention clusters, and 4423 in control clusters. In the intention-to-treat analysis, the incidence of virologically-confirmed dengue was 77% lower in participants who lived in intervention clusters compared to participants who lived in control clusters, and this was similar for all four dengue virus serotypes. In addition, the intervention resulted in an 86% protective efficacy against hospitalisation.

These results demonstrate that release of Wolbachia-infected *A. aegypti* significantly reduces symptomatic disease and hospitalisations in an endemic urban area, showing promise for vector-mediated control of dengue. These results potentially have broader implications for the control of other *Aedes*-borne virus infections, such as those caused by Zika, Yellow fever, or Chikungunya viruses.
